# INTEGRATION OF OBJECTIVE AND SUBJECTIVE EXPERIENCES OF HEALTH IN OLD AGE

**DOI:** 10.1093/geroni/igz038.2288

**Published:** 2019-11-08

**Authors:** Shannon T Mejia, Johanna Drewelies, Cynthia Berg

**Affiliations:** 1 University of Illinois, Urbana-Champaign, Champaign, Illinois, United States; 2 Humboldt University Berlin, Berlin, Germany, Germany; 3 University of Utah, Salt Lake City, Utah, United States

## Abstract

Life-span perspectives on adult development and aging highlight the processes of understanding and acting to support one’s own health and well-being. Within this framework, subjective measures offer insight into the experience of objective measures of health. This symposium brings together four papers that highlight the overlap and discrepancy between subjective and objective measures of health and how these are shaped by individual development in different contexts and phases of adult development over various timescales. Mejía and colleagues consider the intraindividual dynamics of older adults’ perceived and actual fall risk. Using 30 days of subjective and objective balance assessment, they illustrate individual differences in how awareness of fall-risk relates to physical activity on that day. Koffer and Kamarck use data from the Pittsburgh Healthy Heart Project to examine how reactivity of cardiovascular response and affective experience to daily experiences of stress changes with age. Drewelies and colleagues consider how objective aging feels. Using data from the Berlin Aging Study II, they examine how chronological and subjective age are linked to biological age in older adults. Gonzalez and colleagues use self-reported and behavioral data from an in-context biosocial research lab to comment on the theoretical and methodological implications of integrating objective and subjective measures of experience. The discussion by Berg integrates the four papers, highlights their theoretical and methodological contributions, and considers challenges and opportunities for subjective and objective inquiries of older adults’ experiences of health.

